# Retrospective analysis of idiopathic scoliosis medical records coming from one out-patient clinic for compatibility with Scoliosis Research Society criteria for brace treatment studies

**DOI:** 10.1186/s13013-016-0097-4

**Published:** 2016-10-14

**Authors:** Krzysztof Korbel, Łukasz Stoliński, Mateusz Kozinoga, Tomasz Kotwicki

**Affiliations:** 1 Rehasport Clinic, Górecka 30, Poznan, 60-476 Poland; 2 Spine Disorders and Pediatric Orthopaedics Department, University of Medical Sciences, Górecka 30, Poznan, 60-476 Poland

**Keywords:** Idiopathic scoliosis, SRS criteria, Brace treatment

## Abstract

**Background:**

First author attempted to analyse medical records of patients with idiopathic scoliosis for compliance with the Scoliosis Research Society brace studies criteria. A retrospective analysis of medical records of 2705 girls treated from 1989 to 2002 was carried out.

**Methods:**

Age, Cobb, Risser and menarchal status were analyzed for compliance with the Scoliosis Research Society brace studies criteria: a) age ≥10 years, b) Risser 0–2, c) 25–40° Cobb angle, d) no earlier treatment, e) patients before first menses or not more than one year from first menses.

**Results:**

It has been found that 183 girls out of 2705 were ≥10 years old and in the range 25–40° Cobb angle. One hundred two out of 2705 patients revealed eligible for brace effectiveness study according to SRS 2005 criteria. 120 out of 2705 patients revealed eligible for brace brace effectiveness study according to SRS-SOSORT 2014 criteria.

**Conclusion:**

The excluded patients revealed too old or with too significant Cobb angles. This indicates the changing criteria for scoliosis brace treatment over the time. Direct comparison of current results of brace treatment with historical series of cases turns out to be very difficult.

## Background

The initial idea of the work was to evaluate results of Idiopathic Scoliosis (IS) brace treatment performer at one outpatient clinic in the years 1989–2002. First author attempted to analyse medical records of patients with idiopathic scoliosis for compliance with the Scoliosis Research Society 2005 brace studies criteria. In 2015, the SRS-SOSORT Consensus modified those criteria by excluding the 1-year post-menarche limit as the inclusion criterion [1]. The research on the effectiveness of the brace treatment in IS according to different criteria were carried out. In 1995 Nachemson, Peterson [2] as experts of Scoliosis Research Society presented the results of brace treatment for 286 girls followed through the prospective assessment. The inclusion criteria were: a) female sex, b) diagnosed idiopathic scoliosis, c) bone age between 10–15 years, d) single curvature with apex between 8th thoracic vertebra and 1st lumbar vertebra, e) 25–35° Cobb angle [2]. Also in 1995, Olafsson et al published a retrospective assessment of the effectiveness of Boston brace treatment in 64 patients, 61 girls (95.3 %) and three boys (4.7 %). The inclusion criteria were: at least one year to growth completion considering the bone maturity and curvature progression, b) 25–45° Cobb angle, c) progression ≥5° confirmed with X-ray performed in the period of 6 months before brace application, d) no previous treatment, e) minimum two years of observation from the moment of stopping of brace application and at least 18 years of age, f) both sexes, g) diagnosed idiopathic scoliosis [3]. In 2003 Landauer et al published the results of retrospective research of the effectiveness of brace treatment in 62 patients complied with inclusion criteria : a) girls, b) right-sided thoracic idiopathic scoliosis, c) 20–40° Cobb angle, d) brace treatment started in 10–14 years old, e) no first menses, f) minimum two years before expected skeletal maturity [4]. The SRS criteria of effectiveness of SpineCor brace treatment of 2005 in 2007 were applied by Coillard et al [5] for prospective assessment of 493 patients brace treated in the years 1993–2006. Two hundred forty-nine patients (50 %) complied with complete inclusion criteria, 79 of them (16 %) had not finished the treatment and 170 (34 %) constituted the final material [5]. In 2011 Zaborowska-Sapeta et al published the results of effectiveness of Cheneau brace according to SOSORT and SRS guidelines of the patients treated in years 2003–2008 from one outpatient clinic. The study was prospective and the inclusion criteria were following: a) both sexes, b) diagnosed idiopathic scoliosis, c) 20–40° Cobb angle, d) no previous treatment, e) Risser ≥4 at the moment of treatment finishing, f) minimum one year of observation after completion of brace treatment. The inclusion criteria were met by 72 out of 192 patients (37.5 %), 120 patients (62.5 %) were excluded due to: a) skeletally immature and during the treatment = 66 patients, b) non*-*idiopathic form of the disease = 47 patients. Among 72 patients there were: a) 58 girls (73.4 %), b) 21 boys (26.6 %) [6]. In 2015 Kuroki et al [7] published on retrospectively assessed the effectiveness of OMC brace treatment in 31 patients, 29 girls (93.5 %) and two boys (6.5 %) treated in the years 1999–2010. The inclusion criteria were modified SRS criteria: a) diagnosed idiopathic scoliosis, b) age ≥10 years, c) Risser 0–2, d) patients before first menses or no more than one year from first menses, e) 20–40° Cobb angle, with at least two years observation after reaching the bone maturity. The authors modified the SRS criteria by Cobb angle parameter from the value 25–40° to 20–40° invoking the conformity of Weinstein and the associates [7, 8].

### The aim of the study

To find out how many patients treated in one outpatient clinic in the years 1989–2002 for IS with a corrective brace fulfilled the SRS criteria.

### Study design

Retrospective.

## Methods

A retrospective review of the medical records of girls with IS treated in outpatient clinic of Wiktor Dega University Orthopaedic Hospital in Poznan, Poland in years 1989–2002 was carried out. The outpatient clinic’s scope was intended for all types of scoliosis. The patients age, Cobb angle, Risser bone maturity and menses status were recorded from medical charts and available X-ray documentation. The medical reports were analysed in compliance with 2005 SRS criteria of the brace treatment results assessment (inclusion criteria): a) age ≥10 years, b) Risser 0–2, c) 25–40° Cobb angle, d) no earlier treatment, e) patients before first menses or not more than one year from first menses. Exclusion criteria: a) <10 years, b) Risser >2, c) <25° or >40° Cobb angle, d) patients treated earlier, e) patients with first menses >1 year, f) non-idiopathic form of disease [9].

## Results and discussion

The total number of analysed medical records amounted to 2705. The study included only girls. It has been found that 183 girls out of 2705 (6.8 % of the total treated with a brace) were ≥10 years old and in the range 25–40° Cobb angle. However, considering the status of maturation, the number of girls complying with complete SRS criteria amounted to 102 out of 183 (3.8 % of total of the treated): 42 girls with single thoracic curvature, 60 girls with double thoracic and lumbar curvature. The reasons of excluding 81 girls from analysis were: a) first menses >1 year = 18 patients, b) Risser >2 = 24 patients, c) first menses >1 year and Risser >2 = 39 patients. In the final group of 102 girls the mean age at first visit was 12.6 ± 1.7 years, the mean age at the moment of beginning of brace treatment was 12.9 ± 1.7 years.

If the study followed SRS-SOSORT 2015 criteria [1], the study would include 120 girls. Excluded patients show Table 1.

**Table 1:** Table showing the characteristics of the included subjects not respecting the SRS/SOSORT criteria

Localisation	Amount	Cobb angle	Risser	Menarchal	Age
TH	11	30,9 ± 5,1	3,9 ± 0,8	0	14y 5m ± 1,4
TH/LS	13	30,2 ± 5,0	3,8 ± 0,8	0	13 y 9m ± 1,3
TH	11	32,2 ± 5,0	4,7 ± 0,5	11	15 y 6m ±1,2
TH/LS	28	31,9 ± 4,0	4,2 ± 0,8	28	15 y 0m ± 1,7

Age- first visit; TH-main angle; TH/LS-main angle; Menarchal first present >1 year; Risser sign >2

## Conclusions

The review of literature in range of using by authors the criteria for assessment of effectiveness of IS brace treatment shows diversity and variability. In inclusion criteria the following were considered: a) different values of Cobb angle, b) different biological maturity, c) different age, d) different location of curvature, e) different sexes. The retrospective analysis of SI medical documentation from outpatient clinic in Poznan from the years 1989–2002 showed that girls brace treated in majority were older, more matured and presented a bigger Cobb angle in comparison with current SRS criteria of brace treatment studies. If the study followed SRS-SOSORT 2015 criteria [1], the study would include 120 girls. The above analysis confirms the changing criteria applied in non-invasive SI treatment. Considering the fact that brace treatment coincides with most difficult age for a young man which is adolescence as well as specificity of brace treatment 23 h/24 h, and aesthetic discomfort connected with a brace itself, one should agree that the research should be further carried out aiming at determination of factors decisive in result of IS brace treatment, based on IS natural history. Continuous observations and analysis compared to development acceleration will help to determine the most optimal conditions of SI brace treatment. Direct comparison of current results of brace treatment with historical series of cases turns out to be very difficult.
